# A Text Messaging–Based Support Intervention to Enhance Pre-exposure Prophylaxis for HIV Prevention Adherence During Pregnancy and Breastfeeding: Protocol for a Randomized Controlled Trial

**DOI:** 10.2196/41170

**Published:** 2023-01-30

**Authors:** Jerusha Nyabiage Mogaka, Felix Abuna Otieno, Eunita Akim, Kristin Beima-Sofie, Julia Dettinger, Lauren Gomez, Mary Marwa, Ben Odhiambo, Kenneth Ngure, Keshet Ronen, Monisha Sharma, Grace John-Stewart, Barbra Richardson, Joshua Stern, Jennifer Unger, Jenna Udren, Salphine Watoyi, Jillian Pintye, John Kinuthia

**Affiliations:** 1 School of Nursing University of Washington Seattle, WA United States; 2 Kenyatta National Hospital Nairobi Kenya; 3 Department of Global Health University of Washington Seattle, WA United States; 4 School of Public Health Jomo Kenyatta University of Agriculture and Technology Nairobi Kenya; 5 Department of Biostatistics University of Washington Seattle, WA United States; 6 Women and Infants Hospital Providence, RI United States

**Keywords:** pre-exposure prophylaxis, text messaging, text message, mobile technology, PrEP adherence, adherence, prevention, pregnancy, pregnant, breastfeeding, maternal, randomized, RCT, peripartum, patient-provider, postpartum, HIV prevention, SMS, HIV, mHealth, mobile health

## Abstract

**Background:**

Cisgender women in Kenya are at elevated risk of HIV acquisition during pregnancy and post partum. Acute HIV infection during pregnancy and breastfeeding accounts for approximately one-third of all vertical HIV transmissions. The World Health Organization recommends offering oral tenofovir-based pre-exposure prophylaxis (PrEP) to pregnant and postpartum women who are HIV negative but at substantial and ongoing risk for HIV acquisition. PrEP delivery for pregnant and postpartum women is expanding within routine maternal child health clinics in Kenya. However, approximately half of pregnant women discontinue PrEP within 30 days of initiation. Therefore, it is crucial to develop PrEP adherence strategies that enhance support for adherence when peripartum events and health issues pose challenges to sustaining PrEP adherence.

**Objective:**

We are conducting a randomized controlled trial to determine the effect of a bidirectional communication platform named Mobile Solutions for Women’s and Children’s Health (mWACh), which utilizes two-way SMS text messaging between patients and remote nurses to support PrEP adherence and address maternal health concerns in real time during the peripartum period.

**Methods:**

The mWACh-PrEP study is a randomized trial designed to support PrEP adherence during the peripartum period by comparing mWACh-PrEP to the standard of care (ie, in-clinic adherence counseling) among women who are HIV negative and initiating PrEP. Purposive sampling was used to select 5 facilities offering PrEP in antenatal clinics in Kisumu and Siaya Counties, and block randomization will be used to divide participants into groups. Participants in the intervention arm will receive a customized messaging curriculum via SMS text messages targeted toward their particular perinatal stage. The primary outcome, PrEP adherence at 6 months post partum, will be evaluated using a log-binomial regression model, adjusting for imbalanced baseline characteristics. Based on a previous study of directly observed dosing conditions, we will use a hair tenofovir concentration cutoff of 0.038 ng/mg (corresponding to 7 doses/week) as the primary adherence outcome measured at 6 months post partum (binary outcome). Qualitative interviews and cost-effective analyses will be conducted to understand the feasibility, acceptability, and economic impact of the intervention.

**Results:**

Enrollment began in March 2022 and is projected to continue until July 2023, with follow-up through March 2024. The study results are expected to be reported in 2025.

**Conclusions:**

This trial will provide insights into using mobile health to enhance PrEP adherence among pregnant and postpartum mothers. Additionally, the findings will have implications for the use of mobile health technology to improve adherence to other daily medications during the peripartum period.

**Trial Registration:**

ClinicalTrials.gov NCT04472884; https://clinicaltrials.gov/ct2/show/NCT04472884

**International Registered Report Identifier (IRRID):**

DERR1-10.2196/41170

## Introduction

### Background

Young cisgender women in East and Southern Africa have a high incidence of HIV, which persists during pregnancy and the postpartum period, with evidence of an over 2-fold increased acquisition risk during these periods [1,2]. HIV infection among pregnant women also negatively impacts infants. Moreover, acute HIV infections during pregnancy and breastfeeding account for approximately 26% of all vertical HIV transmissions [3,4]. Globally, 150,000 infants were newly infected with HIV in 2019 [5], substantially above the global goal of 20,000 new infections among children by 2020. To protect women and children and eliminate vertical transmission, the World Health Organization (WHO) recommends offering daily oral tenofovir (TFV)-based pre-exposure prophylaxis (PrEP) to pregnant and postpartum women who are HIV negative and at substantial ongoing risk for HIV as part of a comprehensive HIV prevention package [6].

PrEP provision to pregnant and breastfeeding women may prevent primary maternal HIV infections and help eliminate vertical HIV transmission globally. Kenya is a world leader in PrEP implementation for pregnant and postpartum women within maternal and child health (MCH) settings [7,8]. In a study integrating PrEP delivery within antenatal care (ANC) [9], most pregnant Kenyan women at risk of HIV acquisition accepted PrEP when offered during ANC [8]. However, >50% of pregnant women discontinued PrEP within 30 days of initiation [8]. Compared to nonpregnant women, pregnant women more frequently reported side effects, which often overlap with common pregnancy symptoms, as a reason for discontinuing PrEP [8,10,11] and less frequently had detectable tenofovir diphosphate (TFV-DP) levels in dried blood spots (DBS) collected at follow-up visits (52% vs 70%) [12], suggesting suboptimal adherence. Studies among women living with HIV found that motivation for antiretroviral therapy (ART) adherence wanes after breastfeeding cessation when the risk of vertical transmission diminishes [13-15]. In qualitative studies, women's reported motivation for PrEP in pregnancy was driven by the desire to have an infant who is HIV negative [10,11]. Postpartum PrEP adherence may be reduced if a mother perceives the medication is only beneficial to her infant, similar to ART adherence patterns among postpartum women living with HIV [13-15]. Moreover, remembering to take PrEP pills may be difficult with the additional demands of newborn care [16]. PrEP effectiveness depends on adherence [17-19]; therefore, PrEP programs must now focus on improving prevention-effective adherence to maximize reductions in HIV incidence [12].

Mobile health (mHealth) technologies for public health have enormous potential to support HIV prevention and adherence among at-risk populations [20-22], including in Kenya, where cell phone ownership among women is very high at ~90% [23]. SMS text messaging–based interventions have demonstrated effectiveness in increasing adherence to ART among people living with HIV [24-27] and better treatment outcomes among women living with HIV during the peripartum period [28]. More recently, the role of SMS text message–based strategies in supporting PrEP adherence has been investigated among men who have sex with men and transgender women in the United States [29,30]. SMS text message interventions have the potential to improve PrEP adherence and could be used to address issues faced by women using PrEP via real-time texting with a health worker [31-35].

Our team developed a bidirectional communication platform, Mobile Solutions for Women’s and Children’s Health (mWACh), utilizing two-way SMS text messaging between patients and remote nurses to support ART adherence and MCH among peripartum women [36]. The mWACh intervention was feasible, acceptable, and effective at improving selected ANC outcomes, including improving ART adherence among pregnant women initiated on ART and maternal and infant birth outcomes and adherence to contraceptives [37-39]. We adapted mWACh to send PrEP-tailored, Information-Motivation-Behavioral Skills (IMB) Model–based SMS text messages to facilitate adherence among pregnant women who initiated PrEP. In a nonrandomized pre- and posttest pilot, mWACh-PrEP recipients were more likely to persist with PrEP use (42% vs 22%) and to self-report high adherence (73% missed <1 pill/week vs 55%) [31]. Nurses adequately addressed issues raised by participants on PrEP use and side effects via text message.

It is essential to develop PrEP adherence strategies for pregnant women that address PrEP side effects that may overlap with pregnancy symptoms and enhance support for adherence when peripartum life events and health issues pose challenges to sustained PrEP adherence. Therefore, we are conducting a randomized controlled trial (RCT) to determine the effect of mWACh-PrEP on PrEP adherence during pregnancy and the postpartum period.

### Study Objectives

The mWACh-PrEP trial (NCT04472884) aims to improve PrEP adherence among peripartum women using an IMB-informed approach. Our overall goal is to determine the effect of the mWACh-PrEP tool on PrEP adherence during pregnancy and the postpartum period among women at high risk for HIV acquisition who initiate PrEP within ANC and to estimate the cost-effectiveness for potential scale-up. We hypothesize that the mWACh-PrEP tool will improve PrEP adherence among women at risk for HIV, be acceptable to patients and providers, and be cost effective. 

## Methods

### Study Design

We are conducting a nonblinded individual-level RCT comparing 2 arms of PrEP adherence to determine the effect of the mWACh-PrEP tool on PrEP adherence among pregnant women at risk for HIV acquisition initiating PrEP within ANC clinics.

### Study Setting and Population

#### Facility Selection Process

The study is being conducted in 5 public health facilities offering routine MCH services within Kisumu and Siaya Counties in Kenya. The facilities were selected based on high HIV prevalence areas, existing infrastructure, established rapport, and discussions with the Kenyan Ministry of Health and the local government of Kisumu County.

#### Participant Eligibility Criteria

Participating facilities are enrolling 600 pregnant women who are HIV negative (300 in each arm), between 24 and 32 weeks of gestational age (to allow for adequate follow-up during pregnancy), and receiving ANC at MCH clinics. Gestational age is ascertained using the last menstrual period and fundal height measurement, which is part of the routine care for women in this region. Eligibility criteria include HIV and tuberculosis negative, HIV acquisition risk score ≥6 (translating to HIV incidence 7.3 per 100 person-years) [40], initiated PrEP during routine ANC clinic in the participating clinics, ≥18 years, has access to a cell phone, plans to reside in the area for at least 1 year post partum, and plans to receive postpartum and infant care at the study clinic.

#### Randomization Arms

**Figure 1:**
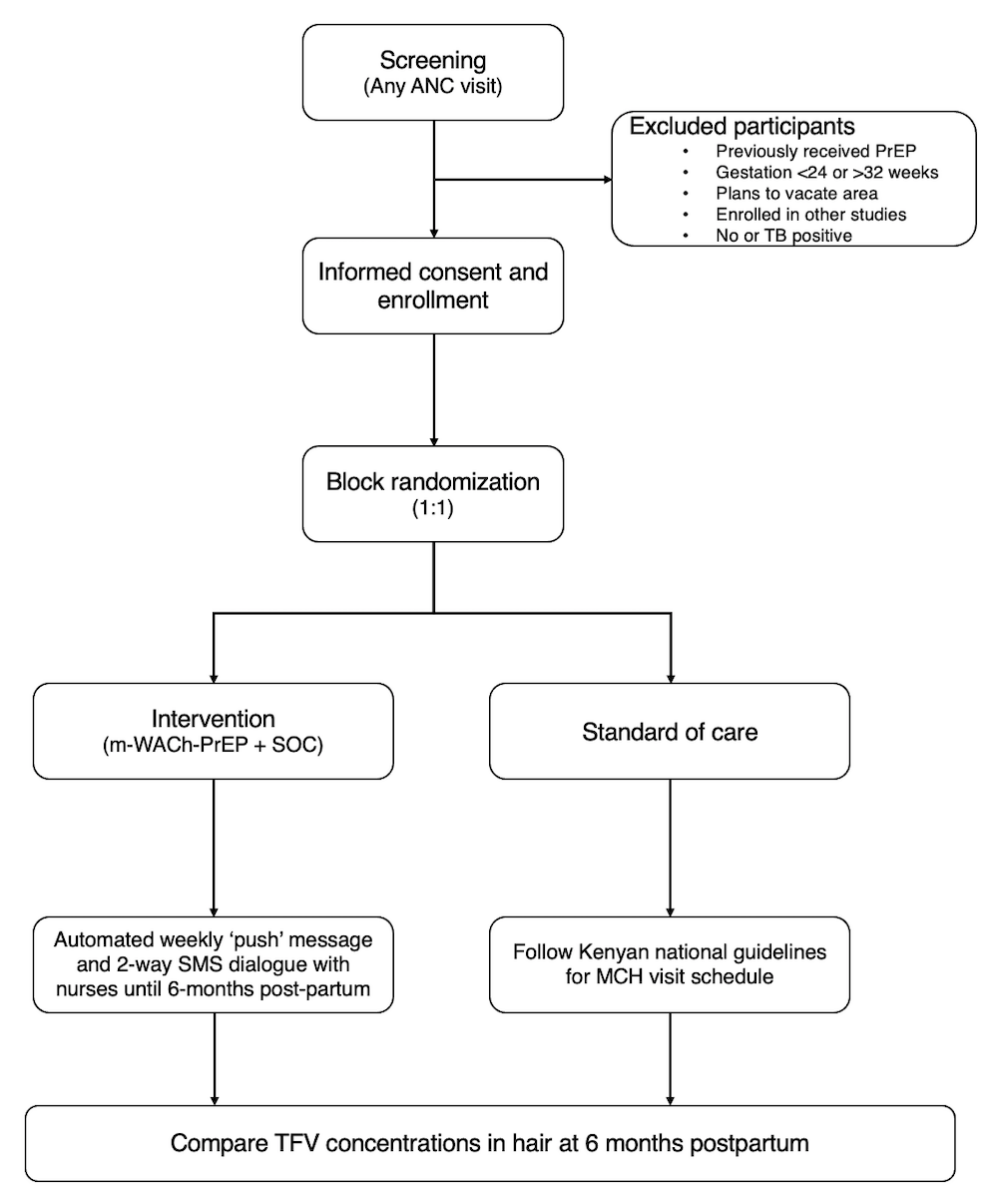
Screening, enrollment, and follow-up procedure for this study. ANC: antenatal care; MCH: maternal and child health; mWACh: Mobile Solutions for Women’s and Children’s Health; PrEP: pre-exposure prophylaxis; SOC: standard of care; TB: tuberculosis; TFV: tenofovir.

Two PrEP adherence models are compared: standard of care (SOC) comprising in-clinic adherence counseling only versus SOC plus the mWACh-PrEP tool. All women receive routine MCH care and PrEP services that include health education, clinical assessments, safety monitoring, screening for sexually transmitted infections (STIs), and treatment per national guidelines (including expedited partner treatment for those with STI diagnoses), HIV risk reduction counseling, assessment of psychosocial barriers, and standard in-clinic adherence counseling per national guidelines. Participants randomized to SOC receive in-clinic counseling on PrEP. Participants randomized to the intervention arm are registered in the mWACh-PrEP system, a bidirectional SMS text message communication platform that sends PrEP-tailored, theory-based, preprogrammed texts on PrEP adherence weekly during pregnancy and the postpartum period, in addition to receiving SOC services (Figure 1).

#### Randomization

Block randomization using a 1:1 allocation in random-sized blocks stratified by recruitment site is being used to ensure a balanced allocation of each arm within sites. Before randomization, study nurses ensure all routine PrEP initiation services and study enrollment procedures are complete. Treatment allocation (mWACh-PrEP or SOC) for each consented participant is done by the study nurses within the individual’s health facility using Research Electronic Data Capture (REDCap; Vanderbilt University). The randomization code is stored in an electronic format, and once assigned, it is unblinded. The randomization key for the database is restricted to the data manager and statistician.

### Study Procedures

#### Main Study

##### Participant Screening and Recruitment

Pregnant women initiating PrEP as part of routine care are approached by the study staff while waiting for ANC and invited to participate in the study. Participants undergo screening, and all eligible participants who express interest undergo an informed consent process at the ANC clinic before the initiation of study procedures. Information on facility volume, clients approached, clients screened, and participants enrolled are reported daily by the study staff. Reasons for nonparticipation are captured from those who decline.

##### Enrollment Visit Data Collection

At enrollment, electronic questionnaires are administered to capture demographic, pregnancy, and medical history; mental health status; HIV results; risk perception; risk assessment; partner characteristics; PrEP attitudes; expected maternity care; health literacy; and COVID-19 experiences. Socioeconomic status is measured using the validated standard Demographic and Health Survey (DHS) Wealth Index [41]. Health literacy is assessed using the standard DHS panel of 8 items across 4 domains (capacity to interpret, obtain, understand, and make appropriate health decisions) [41]. Intimate partner violence (IPV) is assessed using the Hit, Insult, Threaten, Scream scale [42]. Symptoms of depression are assessed using the Edinburgh Postpartum Depression Scale and the Patient Health Questionnaire 9, which has been validated in Kenya [43]. An HIV acquisition risk score validated among pregnant and postpartum Kenyan women is used to assess the risk for HIV [40]. Additional data abstracted from the MCH clinics and PrEP records include details on PrEP initiation, confirmation of syphilis, and HIV test results.

##### SOC Arm Enrollment Visit and Study Procedures

Per national guidelines, all women initiated on PrEP in both arms receive in-clinic counseling on the benefits of sustained PrEP adherence at enrollment and subsequent refill clinic visits, HIV risk reduction, and assessment of psychosocial barriers.

##### Intervention Arm Enrollment Visit and Study Procedures

**Figure 2:**
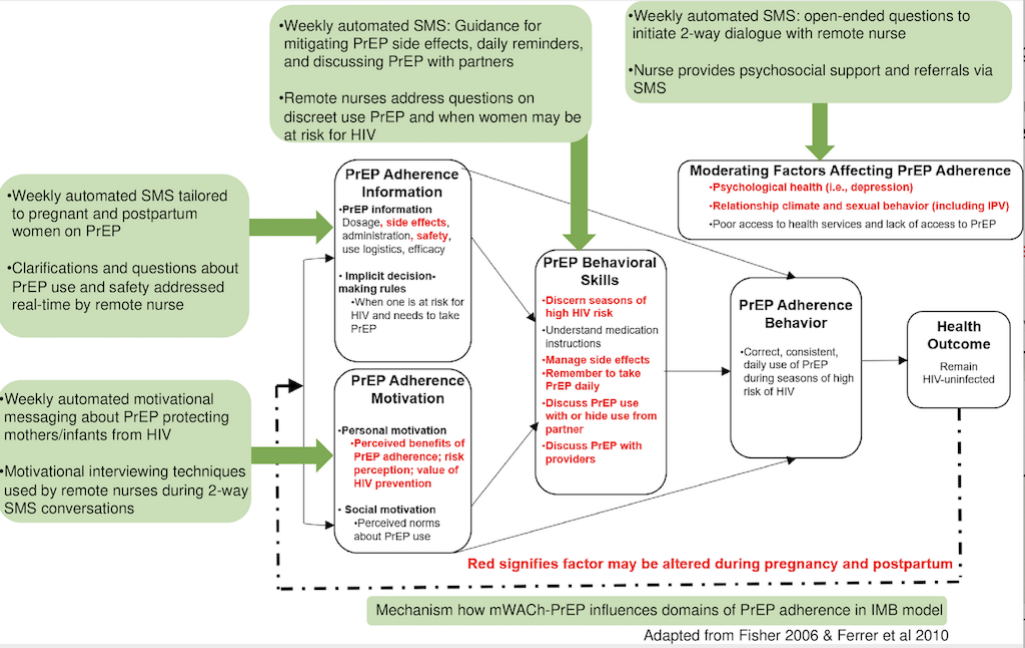
Conceptual model adapted from the Information-Motivation-Behavioral Skills (IMB) Model. mWACh: Mobile Solutions for Women’s and Children’s Health; PrEP: pre-exposure prophylaxis.

**Figure 3:**
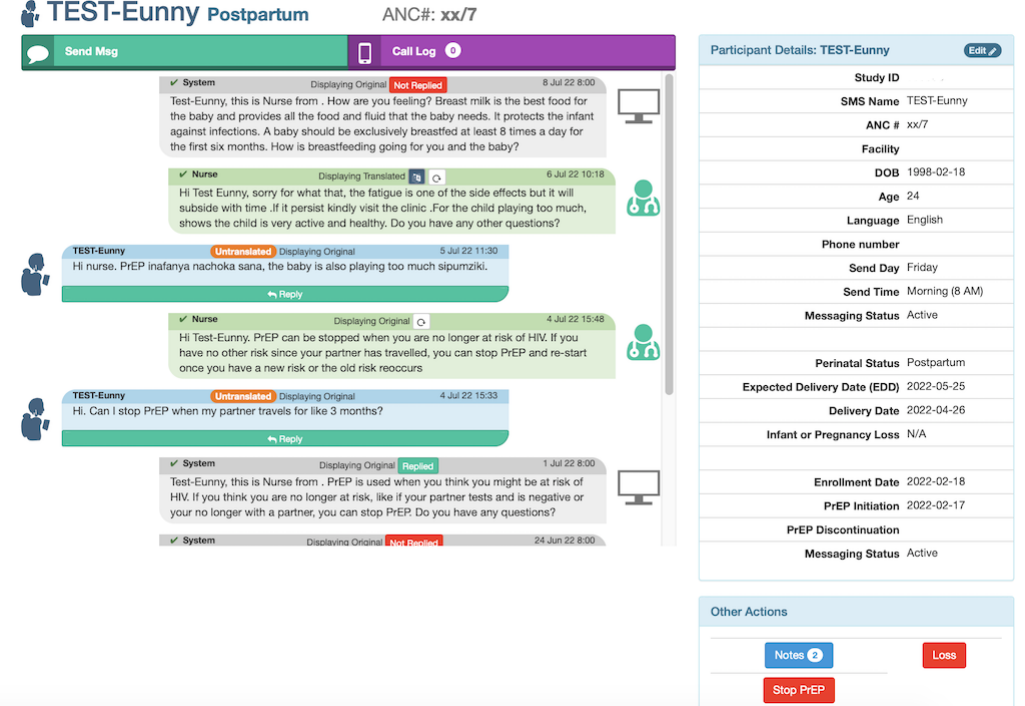
Screenshot of the SMS messaging system. PrEP: pre-exposure prophylaxis.

Participants randomized to the intervention arm are registered in the mWACh-PrEP SMS text messaging system with their preferred name for messaging, messaging language (ie, English, Kiswahili, or Dholuo), and day of the week and time for SMS text message delivery. Once registered, the participants receive weekly automated “push” messages on the day/time of their choice from enrollment until they are 6 months post partum. All automated “push” messages include the participant’s nickname, clinic, and nurse name; an educational message or actionable advice targeting PrEP adherence and continuation and/or MCH topics; and a question related to the content. Through multiple messaging tracks, participants receive messages targeted to their perinatal status as well as supportive messages if they decide to discontinue or restart PrEP. SMS text message topics include adherence encouragement (IMB domain: motivation), PrEP efficacy and safety (IMB domain: information), self-efficacy for prevention of HIV, support for potential PrEP side effects, behavioral skills (eg, tips for remembering PrEP medications; IMB domain: behavioral skills [44,45]), and visit reminders (Figure 2)**.** Participants have the option to respond to automated messages or extemporaneously contact the study nurses at any time. Study nurses encourage participants to respond with concerns and questions (Figure 3). Participants have access to a two-way dialogue with remote nurses until their 6-month postpartum visit. Participants are free to voluntarily exit autonomously by texting “STOP,” thereby ending all communication. All messaging is free to the participant using a reverse-billed short code. The mWACh system also includes multiple features to support study management and personalized counseling based on the participant's psychosocial status (Table 1).

**Table 1:** Example mWACh^a^-PrEP^b^ SMS text messages based on the IMB^c^ model.

Construct		Week	Example messages
**Information**			
	Dosage	2 weeks after PrEP initiation	{name}, this is {nurse} from {clinic}. There are some misconceptions about PrEP in the community. People should not only take the PrEP pill just before potential exposure to HIV. Rather, it should be taken every single day for it to build up in your system and offer protection against HIV. Is there anything else that you have heard about PrEP in the community?
	Side effects	2 weeks after PrEP initiation	{name}, this is {nurse} from {clinic}. A few PrEP users may experience side effects related to PrEP. Most side effects lessen after the first few weeks of use once the body is used to the medication. Please let us know if you are having any side effects. We can help you manage them or know if it is okay to continue.
	Safety	19 weeks after PrEP initiation	{name}, this is {nurse} from {clinic}. PrEP is very safe and one of the ways you can protect yourself from HIV. With this pill, you can keep yourself safer from HIV. Are you still taking the medication? Do you have any questions or concerns?
	Use logistics	4 weeks postpartum	{name}, this is {nurse} from {clinic}. At this time, you may have a lot to deal with as you adjust to having a newborn. Remember to continue taking care of yourself to prevent HIV by consistently taking PrEP. Do you have any questions?
	Efficacy	1 week after PrEP initiation	{name}, this is {nurse} from {clinic}. PrEP is very effective at preventing you from getting HIV. For this effectiveness to be achieved, it is important to take the medication for the first 7 days after initiation without being exposed and thereafter daily. You can use condoms or abstain during this period. Do you have any concerns about PrEP?
**Motivation**			
	Perceived benefits of adherence	4 weeks after PrEP initiation	{name}, this is {nurse} from {clinic}. PrEP is very effective at preventing you from getting HIV if you take it every day. It also helps prevent HIV in your baby if you are pregnant. If you miss too many doses it may not work. Are you having any challenges taking the medication?
	Risk perception	17 weeks after PrEP initiation	{name}, this is {nurse} from {clinic}. PrEP is used when you think you might be at risk of HIV. If you think you are no longer at risk, like if your partner tests and is negative or you are no longer with a partner, you can stop PrEP. Do you have any questions?
	Value of HIV Prevention	15 weeks after PrEP initiation	{name}, this is {nurse} from {clinic}. You are doing a great job taking care of yourself. Deciding to take PrEP is an important way to protect yourself from HIV. It is also important to be tested regularly. Do you have any questions about PrEP?
**Behavioral Skills**			
	Discern seasons of high HIV risk	6 weeks after PrEP initiation	{name}, this is {nurse} from {clinic}. Some women choose to discontinue PrEP for many reasons. It is okay to stop the medication if you decide. We would want to know why people stop PrEP. Have you stopped taking PrEP? If you have why did you stop?
	Remember to take PrEP daily	8 weeks after PrEP initiation	{name}, this is {nurse} from {clinic}. It can be difficult to take medications every day especially if you are trying to be discrete. Many people ask a friend to help remind them, set a timer on their phone, or take it with a meal. You can also put it in a different container you can carry with you in private. How do you remember to take your medication? Do you have any challenges taking it every day?
	Manage PrEP use in relation to partner	9 weeks after PrEP initiation	{name}, this is {nurse} from {clinic}. PrEP enhances the power you have to make an independent decision regarding preventing yourself from HIV as you do not have to rely on your partner for this decision. Do you have any questions about PrEP?
**Moderating Factors**			
	Psychological health	3 weeks after PrEP initiation	{name}, this is {nurse} from {clinic}. Sometimes new mothers can feel sad, anxious, or worried all the time. This happens to many women and can cause crying and difficulty sleeping. Are you having any of these problems?

^a^mWACh: Mobile Solutions for Women’s and Children’s Health.

^b^PrEP: pre-exposure prophylaxis.

^c^IMB: Information-Motivation-Behavioral Skills.

##### Data Collection at Follow-up Visits

Study participants are followed up until 9 months post partum to ascertain sustained PrEP adherence at 3 months after cessation of SMS text messages. All study visits for both arms will be aligned with Kenyan national guidelines for standard ANC and infant immunization visit schedules: monthly visits during pregnancy until delivery and then after delivery at 6 weeks, 14 weeks, 6 months, and 9 months post partum. At each visit, nurses administer questionnaires to assess sexual behavior; infant outcomes; IPV; symptoms of depression; and PrEP attitudes, use, and adherence. PrEP use disclosure is assessed at each follow-up visit by asking participants whether they have disclosed to anyone that they use PrEP and, if so, to whom they disclosed this (Table S1 in Multimedia Appendix 1).

Additional data on infant growth, birth weight, estimated gestation at birth, diagnosed preterm birth, and serial growth are abstracted from medical records. Additionally, *z* scores are calculated using WHO AnthroPlus software. Data on birth outcomes (stillbirth, pregnancy loss, mortality, or congenital anomalies) and birth length are abstracted from the MCH medical record or interview.

### Laboratory Procedures

A combination of red blood cells from DBS and hair samples is used to assess PrEP adherence. A strong correlation between dosing frequency and TFV levels in hair has been demonstrated [46], and significant correlations were also seen between hair, plasma, peripheral blood mononuclear cells, and red blood cell levels (in DBS) of PrEP drugs or metabolites [47,48]. Plasma levels reflect exposure over the past 1 to 3 days [49] and are highly susceptible to intraindividual variation; therefore, measuring hair drug levels is a unique approach to determining drug exposure over a longer period [50,51]. DBS are collected from a finger or heel prick from all mothers and infants at all study visits for future assessment of TFV-DP and emtricitabine triphosphate in red blood cells.

Hair samples will be collected from participants at pregnancy follow-up visits and from both the participants and their babies at postpartum follow-up visits. These hair samples will be analyzed for TFV using validated liquid chromatography-tandem mass spectrometry methods at the University of California San Francisco (UCSF) Hair Analytical Lab (HAL). The UCSF HAL found that TFV concentrations in the hair under directly observed dosing conditions are similar by sex, and the same adherence benchmarks can be used for both women and men [50]. Additionally, hair TFV levels have not been shown to alter in pregnancy [51].

### Data Management

The study staff is trained on data collection using REDCap and communication with participants in the intervention arm using the two-way mWACh-PrEP platform to send, receive, and respond to SMS text messages. Baseline and follow-up data are collected and stored electronically via REDCap, transported via a secure socket layer, and only accessible by authenticated users. Designated study staff generate weekly reports on study progress, performance indicators, adverse events, and troubleshooting problems. All study laptops used to collect data and study databases are encrypted and password protected. All data collected in this study will be available free of charge after registration to access or download files on a study-related website (URL to be determined) after the completion of primary analyses.

### Data Analyses

#### Sample Size

The sample size was calculated to detect a difference between trial arms dependent on the frequency of PrEP adherence in the SOC arm of the cohort. Prior data from our PrEP in pregnancy studies indicate that the frequency of detectable PrEP drug levels is approximately 50% among pregnant women who initiate PrEP in MCH clinics within fully programmatic settings [12]. Since this study will exclusively enroll women who initiate PrEP in MCH, we anticipate that adherence will be similar or slightly higher. Assuming a PrEP adherence estimate of 50% in the SOC arm at 6 months post partum and a sample size of 600 (randomized 1:1), we will have 80% power to detect a minimum detectable difference of 12% between arms in PrEP adherence in the mWACh arm, assuming 15% attrition and a 2-sided test with a Cronbach α of .05 (Table S2 in Multimedia Appendix 1).

#### Statistical Methods and Analyses

The primary outcome, PrEP adherence at 6 months post partum, will be assessed in an intention-to-treat analysis comparing randomization arms mWACh-PrEP versus SOC. Enrollment characteristics will be compared between randomization arms to assess the balance between arms and control for any imbalanced factors. Based on a previous study of directly observed dosing conditions, we will use a hair TFV concentration cutoff of 0.038 ng/mg (corresponding to 7 doses/week) as the threshold of detection for the primary adherence outcome measured at 6 months post partum (detectable/nondetectable binary outcome) [52]. Protective PrEP drug level thresholds for cisgender women have not been established; however, current WHO and Kenyan national guidelines recommend daily (7 doses/week) oral PrEP for cisgender women. A log-binomial regression model will be used to estimate the relative risk, adjusting for any imbalances in baseline demographic, clinical, or behavioral characteristics between arms. Missing primary outcomes or loss to follow-up will be assumed nonadherent. To evaluate the sustained effect of mWACh-PrEP on PrEP-taking habits after the SMS text messaging intervention ends, the same model will be used to analyze our secondary outcome of PrEP adherence at 9 months post partum (3 months after cessation of mWACh-PrEP messaging).

#### Qualitative Analyses

**Figure 4:**
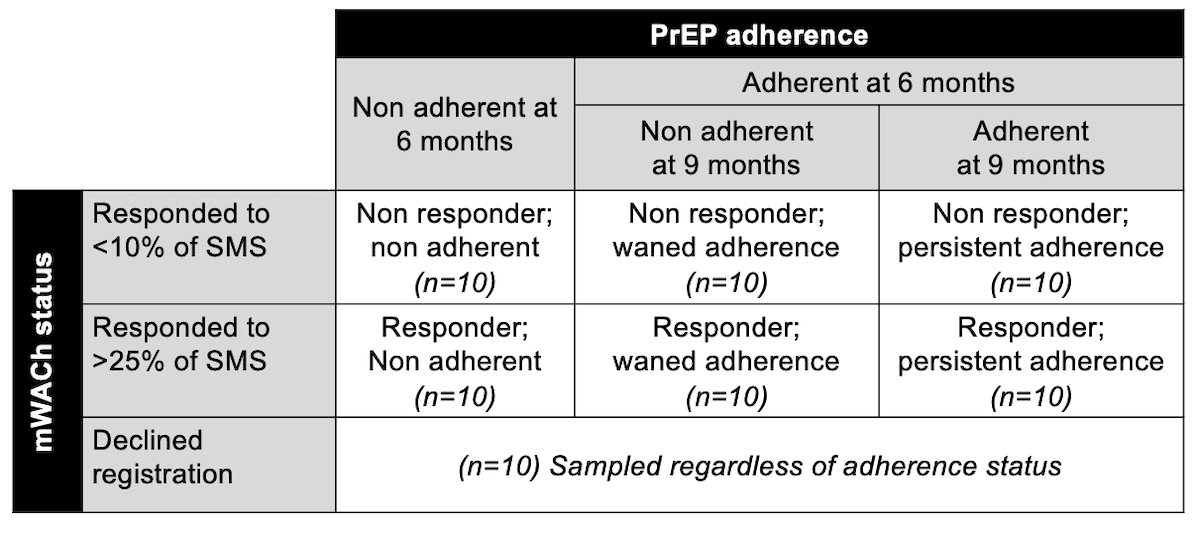
Schematic of qualitative purposive stratified sampling approach (N=70). PrEP: pre-exposure prophylaxis.

Focus group discussions and semistructured in-depth interviews will be conducted to evaluate potential barriers and facilitators to the acceptability and feasibility of mWACh-PrEP implementation within routine ANC delivery settings (Figure 4). To evaluate personal experiences with the mWACh-PrEP intervention, a purposively selected sample of study participants will be recruited to participate in in-depth interviews. In-depth interviews will explore community, dyadic, and individual factors influencing decision-making and how these influence individual perceptions of and experiences with mWACh-PrEP use. To gather perspectives on the mWACh-PrEP scale-up, we will invite the study’s community advisory board (CAB) and county-level health officials overseeing ANC-PrEP programs to participate in focus group discussions. These focus group discussions will elicit perspectives on foreseeable barriers and facilitators of larger-scale implementation of mWACh-PrEP based on individual, community, and health system factors.

Discussions will be conducted in English, Kiswahili, or Luo depending on participants' preferences and be guided by prepiloted topic guides with the flexibility to explore additional probes and newly emerging content as necessary. All in-depth interviews and focus group discussions will be audio recorded, transcribed, and translated to English for analysis as necessary. Thematic analysis will be used to identify key elements of the patient/provider experience, including convenience, comfort level, and satisfaction of the mWACh-PrEP model, that influence intervention acceptability, feasibility, and potential scale-up.

#### Cost-effectiveness Analysis

##### Overview

We will project the cost-effectiveness of implementing mWACh-PrEP intervention within ANC compared to the SOC per averted disability-adjusted life year (DALY) using a payer perspective and collect intervention costs from expense reports, staff, and expert interviews. Costs will be divided into mutually exclusive categories, including personnel, equipment, supplies, buildings and overhead, and start-up. We will convert the local currency, Kenyan shillings, to US dollars. Sensitivity analyses will be conducted to identify influential assumptions.

##### Costing

Time and motion observation of intervention activities will be conducted to calculate staff time and resources necessary for the mWACh-PrEP intervention. A research assistant (RA) will collect data on the time and motion of patient/provider interactions to inform costs and productivity assumptions. The RA will also conduct a survey with health care workers to assess daily responsibilities associated with the mWACh-PrEP intervention, including time to send and respond to client SMS text messages and routine service provision. Research time (eg, administering study questionnaires) and other research costs will be separated from programmatic costs.

##### Modeling

We will parameterize a previously developed mathematical model with cost and outcome data from the mWACh-PrEP trial and project the averted HIV infections, HIV-related deaths, and DALYs associated with scaling up the mWACh-PrEP intervention in Kenya. We will calculate the incremental cost-effectiveness ratio as the difference in costs divided by the difference in effects (DALYs) for the intervention compared to SOC over a 20-year period. Costs and benefits will be discounted annually at 3%.

#### Secondary and Exploratory Analyses

Secondary outcome analyses will include sustained PrEP adherence by determining levels of detectable TFV in the hair at 9 months post partum, adherence cofactors, STI incidence, and prevention-effective adherence (time-varying alignment of adherence with risk behaviors). Exploratory analyses will include HIV incidence, perinatal outcomes, and expedited partner treatment outcomes by randomization arms (Table S3 in Multimedia Appendix 1).

### Ethics Approval

Ethical approval was obtained from the institutional review board of the University of Washington (STUDY00010797) and the Kenyatta National Hospital Ethics and Research Committee (P319.05/2021). This study was registered at ClinicalTrials.gov. (NCT04472884). Any critical protocol deviations, adverse events, and future modifications will be communicated promptly to these organizations. Additional permissions will be sought from all participating clinical sites.

### Informed Consent

A written informed consent before recruitment is administered to all study participants, with each receiving a copy of the signed form. Additional permission will be obtained for audio recording and note-taking during the qualitative interviews.

All participants are informed that their participation is voluntary, and they can stop receiving the SMS text messages at any time by contacting the study staff or texting “STOP,” which will cease communication between the study staff and the participant. All study staff is trained in protecting human participants and the clinical and emotional needs of pregnant and postpartum women on PrEP. The MWACh system is point-to-point encrypted; therefore, only study team members can access it through a password-protected laptop stored in a locked office. All data are deidentified and kept in password-protected databases in a locked study office that is accessible only to study personnel.

#### Patient and Public Involvement

A CAB has been established in Kisumu County to review the study objectives, methodology, and other study activities. The team comprises local community leaders, women from the community, the Kenyan Ministry of Health, and county health representatives from MCH departments. The CAB is anticipated to meet 2 times per year to review this study’s progress. An external advisory panel (EAP) has been established to oversee the data and safety of study participants by reviewing study aims, statistical analysis plan, and progress. EAP meetings will be routinely convened to review study enrollment, retention, data missingness, and pooled study outcomes. Study results will be disseminated through peer-reviewed journals, technical reports, policy briefings, and oral presentations at relevant local and international conferences. Additionally, we will present results to clinical experts and policy advisors at the local county level, the Kenyan Ministry of Health, and the WHO. Routine hybrid meetings in the form of progress updates with CABs engaged in the study activities, facility staff, and study collaborators will be conducted to share study progress and findings.

## Results

Study enrollment began in March 2022 and is projected to continue until July 2023, with patient participant follow-up through March 2024. The study results are expected to be reported in 2025.

## Discussion

### Anticipated Findings and Implications

This intervention study aims to determine the effect of the mWACh-PrEP tool on PrEP adherence among pregnant and breastfeeding women at high risk for HIV acquisition who initiate PrEP during ANC. The primary outcome is adherence at 6 months, while the secondary outcomes include sustained adherence at 3 months after cessation of the SMS text messaging intervention and other HIV and MCH indicators. This is the first intervention that aims to enhance PrEP adherence among women in the peripartum period research, and we anticipate that our findings will inform clinical practice for PrEP delivery during the peripartum period by providing effectiveness data on an mHealth PrEP adherence tool tailored for the peripartum period.

### Study Strengths and Limitations

This is the first intervention study aiming to improve PrEP adherence among peripartum women using an IMB-guided approach tailored to pregnant and postpartum women on PrEP for HIV prevention. The development of adherence strategies for periods during and after pregnancy can have implications for other daily medications taken during pregnancy and the postpartum period when medication side effects overlap with typical symptoms, and life events pose challenges to sustained adherence. Furthermore, collected microcosting data will guide researchers and policy makers on the cost of scale-up and use of mHealth and the IMB-informed approach.

Daily oral tenofovir disoproxil fumarate (TDF)/emtricitabine is currently the only medication with a label indication as PrEP in Kenya and the only regimen recommended by the WHO for cisgender women. While several novel PrEP agents are being tested, including long-acting injectable cabotegravir (CAB-LA) [53], forthcoming results are still years away, and it will take longer for CAB-LA to reach pregnant or lactating women as there is no safety evaluation underway in these populations. Similarly, event-driven PrEP (ED-PrEP) is not approved by the US Food and Drug Administration. Moreover, WHO guidelines explicitly state that ED-PrEP is not appropriate for cisgender women, citing effectiveness concerns in this group [54]. Therefore, daily oral TDF/emtricitabine will likely remain the PrEP SOC for pregnant and breastfeeding women for several years. Because this is a programmatic RCT, the characterization of some secondary and exploratory outcomes will be limited. This design was intentional to optimize program/policy relevance. Costing data will be collected to understand how additional time spent by nurses on the intervention in already busy health facilities may potentially affect scale-up.

### Dissemination Plan

To provide insights on the effects of mWACh-PrEP, we will communicate the study protocol, progress report, and results to the scientific community and policy makers. This will guide the clinical management of pregnant women who choose to initiate PrEP, informing future scale-up of the intervention as a population-level program. Dissemination of the results will potentially improve communication between researchers, ethical bodies, and policy makers and strengthen collaborations between participating partners through technical reports and policy briefings. For the scientific community, we will publish our findings in peer-reviewed journals and present them at international scientific conferences. Presentations and progress reports regarding the study will be shared with study participants and presented to the public at the facility level and to the Kenyan Ministry of Health.

### Conclusion

This study will inform clinical practice and contribute to advancing strategies to enhance PrEP and other daily medication adherence during pregnancy and the postpartum period, which are both critical periods for MCH. If our mWACh approach positively affects MCH outcomes (eg, birth and infant outcomes), it could be readily integrated into other mHealth platforms aiming to improve MCH in the future. The study will also contribute to an mHealth adherence tool for pregnant women on PrEP with the goal of enhancing PrEP use and ultimately reducing HIV incidence in this important population that is disproportionately affected by HIV.

## Appendix

Multimedia Appendix 1 Tables S1-S3.

## Abbreviations

ANC: antenatal care
ART: antiretroviral therapy
CAB: community advisory board
CAB-LA: long-acting injectable cabotegravir
DALY: disability-adjusted life year
DBS: dried blood spots
DHS: Demographic and Health Survey
EAP: external advisory panel
ED-PrEP: event-driven pre-exposure prophylaxis
HAL: Hair Analytical Laboratory
IMB: Information-Motivation-Behavioral Skills
IPV: intimate partner violence
MCH: maternal and child health
mHealth: mobile health
mWACh: Mobile Solutions for Women’s and Children’s Health
PrEP: pre-exposure prophylaxis
RA: research assistant
RCT: randomized controlled trial
REDCap: Research Electronic Data Capture
SOC: standard of care
STI: sexually transmitted infection
TDF: tenofovir disoproxil fumarate
TFV: tenofovir
TFV-DP: tenofovir diphosphate
WHO: World Health Organization

## Data Availability

The data sets generated and analyzed during this ongoing study are available from the corresponding author upon reasonable request.
