# Correction: A Text Messaging–Based Support Intervention to Enhance Pre-exposure Prophylaxis for HIV Prevention Adherence During Pregnancy and Breastfeeding: Protocol for a Randomized Controlled Trial

**DOI:** 10.2196/47124

**Published:** 2023-03-17

**Authors:** Jerusha Nyabiage Mogaka, Felix Abuna Otieno, Eunita Akim, Kristin Beima-Sofie, Julia Dettinger, Lauren Gomez, Mary Marwa, Ben Odhiambo, Kenneth Ngure, Keshet Ronen, Monisha Sharma, Grace John-Stewart, Barbra Richardson, Joshua Stern, Jennifer Unger, Jenna Udren, Salphine Watoyi, Jillian Pintye, John Kinuthia

**Affiliations:** 1 School of Nursing University of Washington Seattle, WA United States; 2 Kenyatta National Hospital Nairobi Kenya; 3 Department of Global Health University of Washington Seattle, WA United States; 4 School of Public Health Jomo Kenyatta University of Agriculture and Technology Nairobi Kenya; 5 Department of Biostatistics University of Washington Seattle, WA United States; 6 Women and Infants Hospital Providence, RI United States

In “A Text Messaging–Based Support Intervention to Enhance Pre-exposure Prophylaxis for HIV Prevention Adherence During Pregnancy and Breastfeeding: Protocol for a Randomized Controlled Trial” (JMIR Res Protoc 2023;12:e41170), the authors noted a typographical error in one of the senior author’s name.

In the originally published article, this author’s name was incorrectly spelled as:

Jilian Pintye

This has now been corrected to:

Jillian Pintye

The correction will appear in the online version of the paper on the JMIR Publications website on March 17, 2023, together with the publication of this correction notice. Since we have not made any changes to the main paper, we have not resubmitted the article.

