# Supplementary material for: A Text Messaging–Based Support Intervention to Enhance Pre-exposure Prophylaxis for HIV Prevention Adherence During Pregnancy and Breastfeeding: Protocol for a Randomized Controlled Trial
Source: JMIR Res Protoc. 2023 Jan 30;12:e41170. doi: 10.2196/41170 (PMC9926344; doi:10.2196/41170)

## Multimedia Appendix 1

#### CONSORT-EHEALTH

**Table S1.** Study timeline for enrollment, randomization, and follow-up visits.

| **Study Period** | | | | | | | | | | | |
| --- | --- | --- | --- | --- | --- | --- | --- | --- | --- | --- | --- |
|  | **Enrollment** | **Randomization** | **Post-allocation** | | | |  |  | |  |  |
|  |  |  | **ANC visit*** | | | | **Postpartum care visits** | | | |  |
| **Timepoint** |  | **0** | ***1*** | ***2*** | ***3*** | ***4*** | ***6 weeks*** | ***14 weeks*** | | ***6 months*** | ***9 months*** |
| Enrollment: |  | | | | | | | | | | |
| Eligibility screen | X |  |  | | | | | | | | |
| Informed consent | X |  |  | | | | | | | | |
| Randomization |  | X |  | | | | | | | | |
| Interventions: |  | | | | | | | | | | |
| *Standard of care* |  |  | X | X | X | X | X | X | X | |  |
| *mWACh-PrEP* |  |  | I | I | I | I | I | I | | I |  |
| Assessments: |  |  |  |  |  |  |  |  | |  |  |
| *Demographic* | X |  | X | X |  | X | X | X | | X | X |
| *Pregnancy* | X |  | X | X | X | X |  |  | |  |  |
| *Medical* | X |  | X | X | X | X | X | X | | X | X |
| *Psychosocial* | X |  |  |  |  |  | X |  | |  | X |
| *Risk assessment* | X |  | X | X | X | X | X | X | | X | X |
| *HIV testing* | X |  | X | X | X | X | X | X | | X | X |
| *Birth and Infant outcomes* |  |  |  |  |  |  |  | X | | X | X |
| *Hair for PrEP adherence* |  |  | X | X | X | X | X | X | | X | X |
| *Number of ANC visits depends on gestational age at enrollment. mWACh-PrEP: short message services intervention, PrEP: pre-exposure prophylaxis, I: intervention arm participants only. X: All study participants | | | | | | | | | | | |

#### Table S2. Sample size, calculations based on various absolute increase in frequency of PrEP adherence, assuming 80% power and alpha =.05.


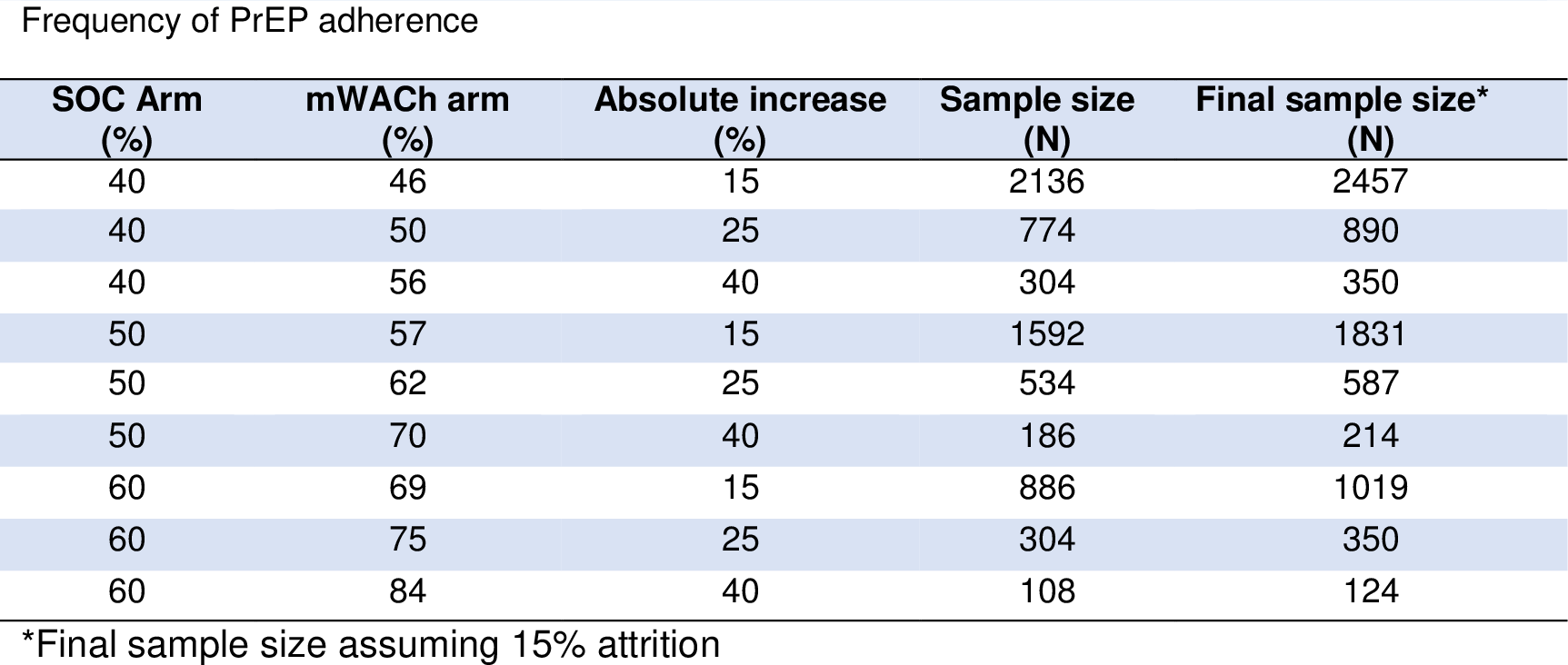


#### Table S3. Secondary and exploratory outcomes of the RCT.


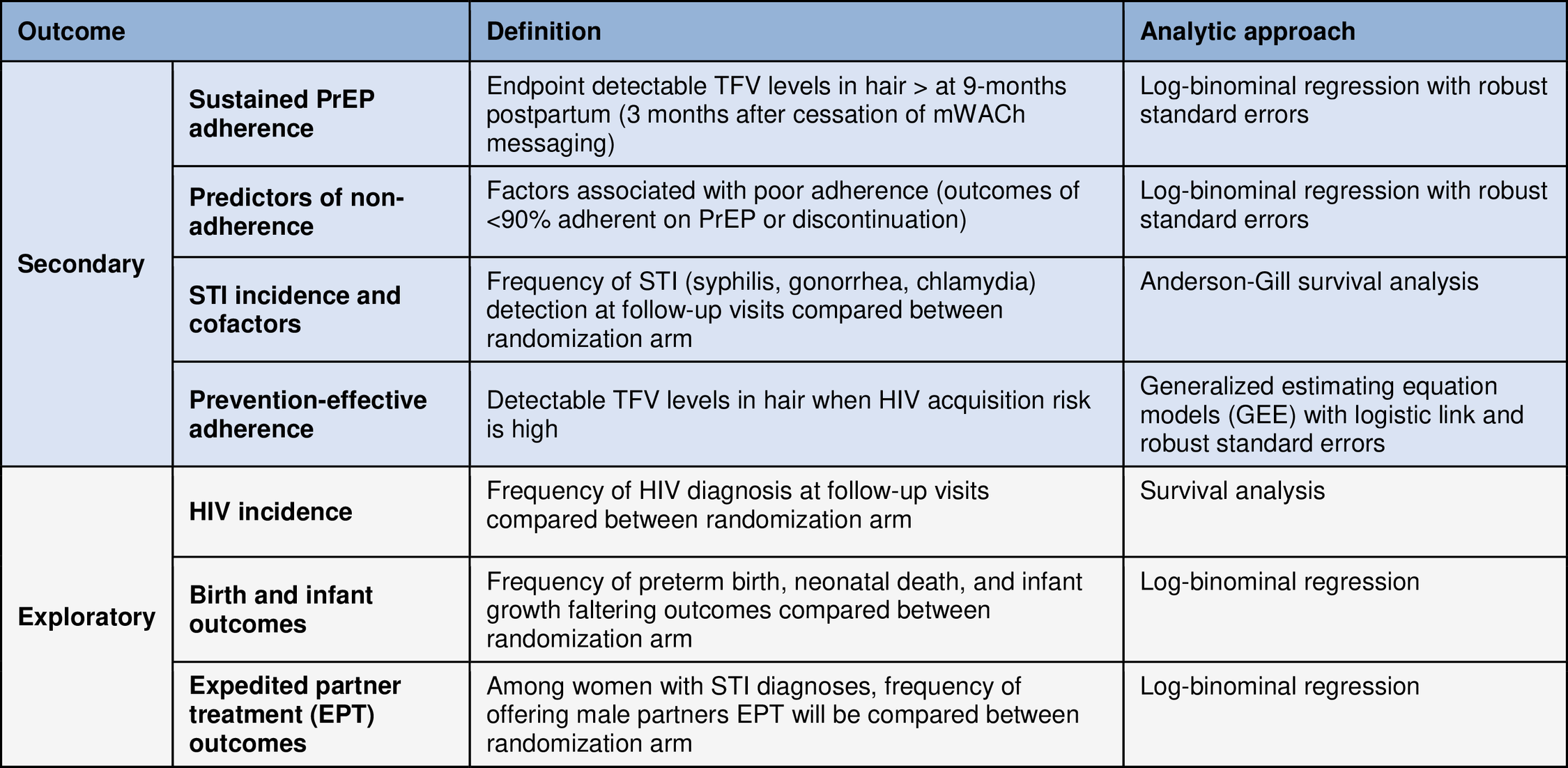

Supplement: Multimedia Appendix 1 [file resprot_v12i1e41170_app1.docx]
